# Association Between Musculoskeletal Pain and Studying Hours Among Medical Students in Jeddah, Saudi Arabia

**DOI:** 10.7759/cureus.45158

**Published:** 2023-09-13

**Authors:** Abdulelah G Abumohssin, Abdulaziz A Alghamdi, Maan A Magboul, Feras W Asali, Mansour S Mahrous, Abdulmajeed A Basaqr, Fahad H Abduljabbar

**Affiliations:** 1 Medicine and Surgery, King Abdulaziz University Faculty of Medicine, Jeddah, SAU; 2 Orthopedics, King Abdulaziz University Faculty of Medicine, Jeddah, SAU; 3 Dermatology, King Abdulaziz University Faculty of Medicine, Jeddah, SAU; 4 Medicine, King Abdulaziz University Faculty of Medicine, Jeddah, SAU; 5 Orthopaedic Surgery, King Abdulaziz University Faculty of Medicine, Jeddah, SAU

**Keywords:** chronic non-specific neck pain, jeddah saudi arbia, low-back pain (lbp), musculoskeletal shoulder pain, musculo- skeletal pain

## Abstract

Introduction

Musculoskeletal (MSK) disorders are one of the common health issues affecting people of various ages. The main risk factors for musculoskeletal pain are age, obesity, gender, level of education, psychosocial factors, occupational factors, decreased mobility and flexibility, and common factors such as consuming TV and video games. College students, especially medical students, are at a higher risk of these complaints as they have longer hours of studying than most faculties due to the competitiveness of their specialty. The objective of this study was to identify the relationship between musculoskeletal pain and studying hours in medical students in Jeddah, Saudi Arabia, and to increase awareness of this problem.

Methods

This cross-sectional study was conducted in 2022. Data was collected using an online questionnaire. A total of 314 participants were included in this study. The demographic variables, studying hours, studying locations, and postures were collected and analyzed.

Results

A total of 314 medical students were included in this study. The majority were males (71.0%) and, the mean age was 22.05±2.13 years. Most of them were sixth-year students, and most of them reported studying between three and four hours (40.1%), with the most common studying location reported being the students' home or residence (79.3%). The number of daily studying hours had no significant effect on the occurrence of musculoskeletal problems.

Conclusion

There was no significant relationship between the number of studying hours and the MSK pain. Clinical trials could be used to evaluate the most effective approaches to alleviate MSK pain in medical students.

## Introduction

Musculoskeletal (MSK) disorders constitute a large portion of the commonest health issues that affect people Across all age groups [1,2]. According to the Global Burden of Disease Study, the prevalence and burden of lower back pain are very high throughout the world [1]. The main risk factors for musculoskeletal pain are age, obesity, gender, level of education, psychosocial factors, occupational factors, decreased mobility and flexibility of muscles, and common domestic factors such as watching TV and computer/video games [3,4]. College students are of major concern as they have to attend lectures, study, and submit projects on a regular basis. Medical students are especially at a higher risk of these complaints as they have longer hours of studying than most faculties due to the competitiveness of their specialty. Algarni et al. conducted a cross-sectional study in Saudi Arabia to determine the incidence of neck, shoulder, and low-back pain among medical students. The study involved 469 medical students using a self-administered online questionnaire. Results of the study showed that more than half of the students complained of MSK pain, especially those in their clinical years, of which some had their normal student lives affected, many of these due to prolonged sitting and awkward postures during lectures and studying [5]. Attar et al. conducted a cross-sectional study aiming to assess the prevalence and risk factors of work-related musculoskeletal disorders (WMSDs) among nursing personnel at a tertiary center in Jeddah. The study involved 200 random nurses and showed a high pattern of WMSDs among nurses in Saudi Arabia (85% of the participants) and a risk factor that contributes to WMSDs was prolonged shifts [6]. Dighriri et al. carried out another cross-sectional study in Jazan, Saudi Arabia, to determine the occurrence of neck, shoulder, and low-back pains among medical students and determine the factors associated with it. The study involved 440 medical students using a self-administered questionnaire. The result of the study demonstrated a substantial prevalence of MSK pain among participants [7].

Haroon et al. conducted a cross-sectional study to evaluate the rate and anatomical distribution of MSK pain and the risk factors related to its occurrence. The study involved 360 medical students. Some factors contribute to MSK pain, such as neck trauma and mental stress. A questionnaire was distributed among the participants. The questionnaire was divided into two parts: MSK pain in the past year and the past week. They found out that 268 of the students had MSK pain during the past week and 140 during the past year. There seems to be an alarming rate of MSK pain among medical students [8].

There is a shortage of research concerning this topic in our region, thus not signifying the importance of this problem among medical students [9]. Our objective in this study was mainly to identify the association between MSK pain and hours spent studying among medical students in Jeddah and to increase the awareness of this problem among medical students in the literature.

## Materials and methods

This research project was a cross-sectional study carried out at the Faculty of Medicine, King Abdulaziz University in Jeddah, Saudi Arabia. It was approved by the institutional review board (IRB) at King Abdulaziz University Hospital (Approval number: 541-22). The aim of the study was to investigate the association between MSK pain and the amount of time medical students spend studying. A self-report questionnaire in the form of a Google form was sent to all medical students in the basic and clinical years (2nd, 3rd, 4th, 5th, and 6th years). The data collection period was from November 15, 2022, to January 23, 2023, and the questionnaire was shared with the students through a link on social media sites.

The questionnaire was reviewed by two orthopedic consultants at King Abdulaziz University (KAU) and validated by the Unit of Biomedical Ethics. It comprised 30 questions and was divided into five sections. The first section aimed to gather sociodemographic data, such as gender, weight, height, and school year. The second section aimed to collect information about the participants' studying routine, including the average number of daily studying hours, the location where they studied, the position they adopted while studying, and the comfort of their study furniture. The third section focused on the participants' exercise routine, asking if they exercise, the type of exercise they perform, and whether they stretch before exercising (such as swimming, walking, running, and weight lifting). The fourth section used the validated Nordic Back Pain Questionnaire in English to collect information on MSK pain experienced in the past 12 months in specific body regions, such as neck, shoulders, elbows, upper and lower back, wrists/hands, hips/thighs, knees, and ankles/feet. It also asked whether participants sought medical attention for pain and whether it affected their ability to perform daily activities, with regard to the anatomical sites previously listed. The fifth section consisted of two yes/no questions, inquiring whether the participants suffered from any physical trauma or were diagnosed with rheumatological diseases.

For statistical analysis, Statistical Package for the Social Sciences (SPSS), (IBM Corp., Armonk, USA) was used; continuous variables were described by calculating the mean and standard deviation, while categorical variables were described by numbers and percentages, and the chi-square test was used to evaluate the difference between the continuous and qualitative variables, respectively. A p-value of <0.05 was considered significant.

## Results

Sociodemographic characteristics and studying information

Our study targeted medical students in Jeddah, Saudi Arabia. In order to measure our sample size, we needed to determine the total number of medical students in each university in Jeddah and sum them to get the population size. We contacted each faculty of medicine here in Jeddah to obtain the maximum number of students they accept each year and if it applied to every medical year from the 1st year of medicine until the 6th year according to our medical education system in Saudi Arabia. Accordingly, the number we came out with was around 4000, so we rounded it up to 4000. The confidence interval we wished to attain was 95%, with a margin of error of 5%. The sample size with this criterion was calculated using Raosoft (Raosoft, Inc., Seattle, USA) and was equal to about 350 medical students. For our study to be more accurate, we had exclusion criteria that removed any possible cofounders. Unfortunately, it was at the expense of our sample size, which was reduced from the total we gathered, which was 370, down to 314. This is the limitation that we faced after collecting the data. Males (71.0%) were the majority and the mean age was 22.05±2.13 years. Most of the students were from the 6th year. Table 1 demonstrates the sociodemographic characteristics of the participants

**Table 1:** Sociodemographic characteristics Notes: Age data are expressed as mean ± standard deviation, and others are expressed as numbers and percentages. SD: standard deviation, BMI: body mass index

Sociodemographic characteristics		
	Number	Percentages
Gender		
Male	223	71.0
Female	91	29.0
Medical year		
Second year	56	17.8
Third year	42	13.4
Fourth-year	55	17.5
Fifth year	46	14.6
Sixth year	115	36.6
	Mean	SD
Age (years)	22.05	2.13
BMI	25.68	12.45

Most of the participants reported studying 3-4 hours daily (40.1%), with the most common studying location reported being the students’ home or residence (79.3%). Students were asked about their most favorable position while studying. The most common position reported (68.2%) was “sitting upwards on a chair”. Table 2 demonstrates the students' report concerning daily studying hours, favorable position, and location when studying.

**Table 2:** Information concerning studying

Information concerning studying		
	Number	Percentages
Number of daily study hours		
Less than 1 hour	12	3.8
1-2 hours	33	10.5
3-4 hours	126	40.1
5-6 hours	89	28.3
More than 6 hours	54	17.2
Studying position		
Sitting upwards on a chair	214	68.2
Sitting on the ground	24	7.6
Standing up and walking around	23	7.3
Laying on the back	30	9.6
Laying on the front	13	4.1
Sitting on a bed or a sofa	7	2.2
Don’t have a specific position (switches)	3	1.0
Studying location		
At home	249	79.3
At the university library	26	8.3
At a coffee shop	39	12.4

Musculoskeletal problems reported by the students

In our study, we assessed and categorized different MSK problems into either a problem that occurred over the last seven days or problems that occurred over the last twelve months (any pain, discomfort, or numbness was considered an MSK problem). Lower back pain and neck pain were the most reported problems in the last seven days respectively (N=140, 44.6% and N=126, 40.1%). Similarly, the most reported problems in the last twelve months were also lower back pain (N=129, 41.1%) and neck pain (N=121, 38.5%). The majority had at least one MSK problem in the last seven days and in the last twelve months (83.4% and 80.6%). MSK problems affected the normal activity of the students such as attending the university and practicing hobbies in 20.7% of the cases. Moreover, 17.2% of the participants needed to visit a doctor concerning these problems. In our study, the number of daily hours spent studying did not have any noticeable effect on the development of MSK problems. The location of studying had a significant effect on the occurrence of MSK problems in the last twelve months, as the rate of problems was the lowest among students who studied in their homes, followed by those who studied in the university’s library, and reached its peak in students who study in coffee shops (77.9% vs 84.6% vs 94.9%, p=0.039). Moreover, students who studied standing up and walking around had higher rates of problems (91.3%). However, when comparing the rates of different studying positions, there was no significant relationship between this group of students and the rest (p=0.173).

Table 3 demonstrates the relationships between daily studying hours and the occurrence of pain in different MSK parts.

**Table 3:** The relationships between daily studying hours and the occurrence of pain in different musculoskeletal parts.

	Less than 1 studying hour	1-2 studying hours	3-4 studying hours	5-6 studying hours	More than 6 studying hours	P-Value
Neck pain in the last 12 months						
No	6 (3.1%)	21 (10.9%)	74 (38.3%)	55 (61.8%)	37 (19.2%)	0.689
Yes	6 (5.0%)	12 (9.9%)	52 (43.0%)	34 (28.1%)	17 (14.0%)	
Shoulders pain in the last 12 months						
No	5 (2.2%)	26 (11.5%)	98 (43.2%)	59 (26.0%)	39 (17.2%)	0.052
Yes	7 (8.0%)	7 (8.0%)	28 (32.2%)	30 (34.5%)	15 (17.2%)	
Upper back pain in the last 12 months						
No	10 (4.2%)	29 (12.2%)	91 (38.2%)	63 (25.5%)	45 (18.9%)	0.155
Yes	2 (2.6%)	4 (5.3%)	35 (46.1%)	26 (34.2%)	9 (11.8%)	
Elbows pain in the last 12 months						
No	12 (4.1%)	31 (10.6%)	117 (39.9%)	81 (27.6%)	52 (17.7%)	0.776
Yes	0 (0.0%)	2 (9.5%)	9 (42.9%)	8 (38.1%)	2 (9.5%)	
Lower back pain in the last 12 months						
No	10 (5.4%)	20 (10.8%)	70 (37.8%)	56 (30.3%)	29 (15.7%)	0.317
Yes	2 (1.6%)	13 (10.1%)	56 (43.4%)	33 (25.6%)	25 (19.4%)	
Hands/Wrists pain in the last 12 months						
No	10 (3.9%)	27 (10.5%)	103 (39.9%)	72 (27.9%)	46 (17.8%)	0.984
Yes	2 (3.6%)	6 (10.7%)	23 (41.1%)	17 (30.4%)	8 (14.3%)	
Hip/Thighs pain in the last 12 months						
No	11 (4.0%)	29 (10.5%)	111 (40.1%)	74 (26.7%)	52 (18.8%)	0.185
Yes	1 (2.7%)	4 (10.8%)	15 (40.5%)	15 (40.5%)	2 (5.4%)	
Knees pain in the last 12 months						
No	10 (4.0%)	29 (11.6%)	99 (39.6%)	71 (28.4%)	41 (16.4%)	0.757
Yes	2 (3.1%)	4 (6.3%)	27 (42.2%)	18 (28.1%)	13 (20.3%)	
Foot/Ankles pain in the last 12 months						
No	12 (100%)	30 (90.9%)	109 (86.5%)	72 (80.9%)	47 (87.0%)	0.412
Yes	0 (0.0%)	3 (9.1%)	17 (13.5%)	17 (19.1%)	7 (13.0%)	

Relationships with sociodemographics

The results of our study did not demonstrate any significant relationships between the occurrence of MSK problems and either age, gender, BMI, or medical year as the rates of the problems were similarly present in all the groups.

## Discussion

MSK pain is a worldwide phenomenon that afflicts many people based on their work [10]. Combating this issue may prove to be a major stepping stone in improving the general well-being of the affected individual [11]. There have been, to the best of our knowledge, limited studies done to assess the prevalence of MSK pain among undergraduate medical students in Saudi Arabia. A study done on medical students in the central region of Saudi Arabia reported that 85.3% of the participants had MSK pain in one or more body sites at any time [5]. All the research analyzed in our study stated that participants exhibited MSK symptoms in at least one area of their bodies, with pain being the most prevalent symptom. However, other MSK symptoms were also reported, including muscular weakness and numbness, burning, and tingling [12]. Low back pain (44.6%) and neck pain (40.1%) were the most frequently reported complaints among students in the past twelve months in our study, which is similar to a conclusion reached by a study performed in Al-Qassim in 2021 [13]. In another study done in Saudi Arabia, more severe symptoms were encountered based on the duration of working hours [14]. However, our study showed that there was no significant correlation between studying hours, which constitute a student’s workload, and MSK pain. Moreover, MSK problems were reported regardless of the student’s studying hours, indicating that studying hours may not have been an attributable cause for their discomfort.

Our study shows that students who study standing up and walking around have the highest rate of MSK pain (91.3%). However, compared to the rest, no statistical significance was established between the studying position and the incidence of MSK pain. On the contrary, a study conducted for the assessment of the prevalence and causes of back pain amongst Nigerian adolescents found that prolonged sitting was the strongest risk factor for the development of back pain [15]. Another study performed on school students in South Africa indicated that the degree of postural angulation in particular head flexion posed a significant probability in the occurrence of seated-related upper musculoskeletal pain [16]. The absence of a clear relationship between studying position and MSK pain in medical students could be attributed to the long hours they spend studying continuously, as they are more likely to utilize different positions during these periods and are unable to single out a particular position as the root of their MSK pain. 

The demographic factors in our study, including age, sex, and BMI, also show no statistical significance towards the development of MSK pain in medical students; this is similar to what Algarni et al. reported in their study conducted among students at medical schools in the central region of Saudi Arabia [5]. In contrast, a systematic review showed a strong correlation between a higher BMI and the development of MSK pain [17]. Old age and the female gender also showed a strong correlation with MSK pain, as described by Waersted et al. [18]. Since our study population is comprised entirely of young adults with similar BMIs and levels of activity, demographic factors are unlikely to vary much and thus have no major impact on the development of MSK pain.

Limitations to our studies were mainly due to the cross-sectional nature of the study. We were unable to determine which sequence of events afflicted the study population first - MSK pain or the number of study hours. Also, we cannot exclude the possibility of the study population having recall bias.

## Conclusions

Our study suggests that MSK pain is a common complaint experienced by undergraduate medical students in Jeddah. Based on the results, lower back pain and neck pain were the most prevalent complaints among our participants. However, our findings indicate that no significant relationship exists between the number of study hours and the possibility of MSK disorders manifesting as physical symptoms. It can be assumed that the physical symptoms of MSK disorder may be a detriment to the quality of studying. More detailed studies should be conducted to investigate further the potential of such disorders to influence medical students' continued pursuit of knowledge. Clinical trials could also be used to evaluate the most effective approaches to alleviating MSK pain in medical students.
